# Acoustic and Semantic Processing of Auditory Scenes in Children with Autism Spectrum Disorders

**DOI:** 10.1007/s10803-023-05924-9

**Published:** 2023-05-04

**Authors:** Breanne D. Yerkes, Christina M. Vanden Bosch der Nederlanden, Julie F. Beasley, Erin E. Hannon, Joel S. Snyder

**Affiliations:** 1 Department of Psychology, University of Nevada, Las Vegas, Las Vegas, NV USA; 2 Department of Psychology, University of Toronto Mississauga, Mississauga, ON Canada; 3 Ackerman Center for Autism and Neurodevelopment Solutions, Kirk Kerkorian School of Medicine, University of Nevada, Las Vegas, Las Vegas, NV USA

**Keywords:** Autism spectrum disorders, Acoustic, Semantic, Speech-in-noise, Change deafness

## Abstract

**Purpose**: Processing real-world sounds requires acoustic and higher-order semantic information. We tested the theory that individuals with autism spectrum disorder (ASD) show enhanced processing of acoustic features and impaired processing of semantic information. **Methods**: We used a change deafness task that required detection of speech and non-speech auditory objects being replaced and a speech-in-noise task using spoken sentences that must be comprehended in the presence of background speech to examine the extent to which 7–15 year old children with ASD (n = 27) rely on acoustic and semantic information, compared to age-matched (n = 27) and IQ-matched (n = 27) groups of typically developing (TD) children. Within a larger group of 7–15 year old TD children (n = 105) we correlated IQ, ASD symptoms, and the use of acoustic and semantic information. **Results**: Children with ASD performed worse overall at the change deafness task relative to the age-matched TD controls, but they did not differ from IQ-matched controls. All groups utilized acoustic and semantic information similarly and displayed an attentional bias towards changes that involved the human voice. Similarly, for the speech-in-noise task, age-matched–but not IQ-matched–TD controls performed better overall than the ASD group. However, all groups used semantic context to a similar degree. Among TD children, neither IQ nor the presence of ASD symptoms predict the use of acoustic or semantic information. **Conclusion**: Children with and without ASD used acoustic and semantic information similarly during auditory change deafness and speech-in-noise tasks.

Autism spectrum disorder (ASD) is a class of neurodevelopmental disorders characterized by abnormalities in social interaction and communication, restricted and repetitive behaviors, and atypical sensory processing (American Psychiatric Association, 2013). Past studies have led to the assumption that a unique hallmark of ASD is the combination of enhanced low-level acoustic processing of pitch (Bonnel et al., 2003; Heaton, 2005; Heaton et al., 1999, 2008; Jarvinen-Pasley & Heaton, 2007; Jarvinen-Pasley, Pasley et al., 2008; Jarvinen-Pasley, Peppe, et al., 2008; Mayer et al., 2016; O’Riordan & Passetti, 2006) with impaired higher-order semantic processing (Eberhardt & Nadig, 2018; Frith & Snowling, 1983; Happé, 1997; Jolliffe & Baron-Cohen, 1999; Lopez & Leekam, 2003; Norbury & Bishop, 2002; Tager-Flusberg & Anderson, 1991) in auditory and language tasks. The purpose of the current study is to further test this assumption using measures of auditory segregation of speech and non-speech sounds that have not been examined in past studies of ASD.

Two relevant theories have been put forth to explain sensory and cognitive processing abnormalities in ASD: Weak Central Coherence (WCC) (Frith & Happe, 1994; Happe, 1999; Happe & Frith, 2006) and Enhanced Perceptual Functioning (EPF) (Mottron et al., 2006). The WCC theory postulates individuals with ASD possess a detail-focused cognitive style that involves increased attention to low-level perceptual information accompanied by a diminished ability to integrate individual elements into a coherent whole. This processing style can lead to a reduction in sensitivity to global features and the underuse of contextual information. WCC would predict that compared with typically developing (TD) individuals, those with autism would exhibit enhanced performance on tasks that entail increased attention to local perceptual features, and diminished performance on tasks that involve contextual integration or the use of global information. Like WCC, the EPF (Mottron et al., 2006) model also postulates enhanced processing of simple, low-level perceptual information. However, unlike WCC, this low-level enhancement does not necessarily give rise to impairment in global processing. Instead, EPF assumes that too much activity in low-level sensory brain areas drives enhanced low-level processing. Additionally, EPF posits that relative to TD individuals, individuals with ASD have less interaction between perceptual and higher-order processes, especially during tasks in which it would be beneficial to focus on low-level information. Thus, both WCC and EPF theories would likely predict greater use of low-level rather than semantic information in ASD compared to TD participants because excessive processing of details might detract from processing higher-level meaning of sensory information. The main goal of the current study is to measure the use of semantic information in those with ASD in comparison to TD participants, using auditory segregation tasks that use naturalistic speech and non-speech sounds.

Our daily auditory environment surrounds us with semantically meaningful speech and non-speech sounds. Accurate comprehension of semantically meaningful sounds requires the activation of higher-order information such as semantic context and prior knowledge of sound categories and schemas. For instance, TD listeners process semantically predictable sentences (e.g., The farmer harvested his *crop*) differently than they process less predictable sentences (e.g., I went to a play about a *crop*) (Morgan et al., 2020). Similarly, listeners more readily identify semantically meaningful non-speech sounds that are embedded in a contextually incongruent auditory scene (e.g., a rooster crowing in a hospital) than a contextually congruent scene (e.g., rooster crowing in a farm; Gygi & Shafiro, 2011; Leech et al., 2009). Thus, in both situations, the overall semantic context provides high-level information that enables comprehension of the sentence or auditory scene, and in the case of contextual incongruence, acts as an attentional spotlight on items that conflict with the semantics of the scene.

Children with ASD typically perform worse than TD children on tasks that require them to use sentence context to infer the appropriate pronunciation of a visually presented homograph (e.g., *entrance* meaning a way in to a room, or *entrance* meaning to hypnotize) or to interpret an ambiguous sentence (Frith & Snowling, 1983; Happé, 1997; Jolliffe & Baron-Cohen, 1999; Lopez & Leekam, 2003). Other paradigms have revealed impaired use of semantic context in individuals with ASD (Eberhardt & Nadig, 2018; Norbury & Bishop, 2003; Tager-Flusberg & Anderson, 1991). Relatively few studies of ASD have investigated the use of semantic information during the processing of non-speech sounds. One study found that while individuals with ASD failed to exhibit a neural detection response for semantic incongruencies in speech (N400), their neural responses to semantically incongruous environmental sounds were comparable to TD individuals (McCleery et al., 2010). A separate study found that while TD individuals were equally good at using a semantic prime that was a picture or word, individuals with ASD performed better with a picture than with a word prime (Kamio & Toichi, 2000). Together, these studies suggest that despite deficits of semantic processing in the context of speech and language, processing of semantically meaningful non-speech stimuli is relatively unimpaired in individuals with ASD.

Low-level acoustic information is also important during the processing of semantically meaningful speech (Cutler & Clifton, 1984; Nakatani & Schaffer, 1978) and environmental sounds (Gygi et al., 2007). Studies using pairs of pure tones, or pairs of words and short sentences, often reveal superior discrimination performance in individuals with ASD relative to TD individuals (Bonnel et al., 2003; Heaton, 2005; Heaton et al., 1999, 2008; Jarvinen-Pasley & Heaton, 2007; Jarvinen-Pasley, Pasley et al., 2008; Jarvinen-Pasley, Peppe, et al., 2008; Mayer et al., 2016; O’Riordan & Passetti, 2006). However, the processing of other local features like loudness (Bonnel et al., 2010; Jones et al., 2009; Khalfa et al., 2004) and timing (Falter et al., 2012; Isaksson et al., 2018) does not always show enhanced processing in ASD. Additionally, some evidence suggests that enhanced pitch processing is only present in a subgroup of individuals with ASD who have a history of language delay or language impairments (Bonnel et al., 2010; Heaton et al., 2008; Jones et al., 2009). Even though some individuals with ASD show enhanced pitch processing, they nevertheless can have difficulty perceiving speech in noisy environments (Alcantara et al., 2004, 2012; Bhatara et al., 2009; DePape et al., 2012; Groen et al., 2009). For example, relative to TD controls, individuals with ASD are less able to take advantage of temporal dips or brief pauses in background noise to improve their performance on a speech-in-noise task (Alcantara et al., 2004; Groen et al., 2009). Individuals with ASD also have been shown to have poorer gap detection than TD individuals (Bhatara et al., 2013; Boets et al., 2015; Foss-Feig et al., 2017).

As described above, much of the prior ASD research on semantic context has used visually presented individual words and pictures. Studies of acoustic processing have likewise presented individual sounds (e.g., pure tones and speech), or sounds with noise. To our knowledge, no studies have asked how TD listeners and those with ASD use acoustic and semantic information when presented with multiple, simultaneous, semantically meaningful non-speech sounds during segregation tasks. We used change deafness and speech-in-noise tasks to examine how listeners use semantic information when segregating natural sounds in auditory scenes. A novel aspect of our approach compared to past studies of ASD is that, whereas prior studies examined speech comprehension in quiet situations, the speech-in-noise task allows us to examine how semantic information is used during segregation of speech from background noise. The change deafness task we use is even more novel because it requires segregation of non-speech sounds that are nevertheless meaningful, which has not been studied in ASD to our knowledge.

Change deafness is an auditory phenomenon, analogous to change blindness in the visual domain (Simons & Rensink, 2005; Snyder & Gregg, 2011; Snyder et al., 2012), where salient auditory changes go unnoticed by listeners in the presence of an array of other simultaneous sounds. Acoustic changes in pitch and harmonicity that are small (e.g., Chihuahua bark changing to a chicken clucking) are more difficult to detect than changes that are larger (e.g., Chihuahua bark changing to hands clapping; Gregg & Samuel, 2008, 2009; Vanden Bosch der Nederlanden, Snyder, & Hannon, 2016). Changes to an incongruent semantic category (e.g., Chihuahua bark changing to a trumpet) are easier to detect than changes to the same semantic category (e.g., Chihuahua bark changing to a Great Dane bark) (Gregg & Samuel, 2009; Vanden Bosch der Nederlanden et al., 2016). Similarly, listeners find it easier to identify sounds that are semantically incongruent with a background scene (e.g. a barn scene) than sounds that fit with that scene (Leech et al., 2009). Additionally, changes involving the human voice are detected better than changes that involve other semantic categories (environmental, musical, animal) (Vanden Bosch der Nederlanden et al., 2020), suggesting there is an attentional bias toward certain categories of semantically meaningful vocal human sounds over others (e.g., communicative vs. non-communicative; sneezing vs. laughing). One prediction that follows from both EPF and WCC is that individuals with ASD would be less likely to exhibit change deafness due to their tendency to focus on low-level features and ignore high-level semantic information. Furthermore, given social deficits in ASD we might also expect that compared with TD listeners, those with ASD might show less of an advantage for detecting changes involving human voices.

The role of semantic context can also be examined in a speech-in-noise task by using sentences with high- and low-predictability at different signal-to-noise ratios (SNR). An example of a sentence with high predictability is “The candle flame melted the *wax*” where the semantic context, specifically the words “candle”, “flame”, and “melted” assist in predicting the last word, “wax”. An example of a sentence with low predictability is “Paul can’t discuss the wax.” High predictability sentences are more accurately perceived than low predictability sentences, especially at lower SNR’s, presumably because listeners use semantic information to aid their speech-in-noise processing (Bradlow & Alexander, 2007; Kalikow et al., 1977; Pichora-Fuller et al., 1995; Wilson et al., 2012).

The current study used change deafness and speech-in-noise tasks with TD and ASD children, primarily to study how much TD and ASD children use semantic information to perceptually segregate natural speech and non-speech sounds. We chose the change deafness task because it allowed us to separately examine children’s use of acoustic and semantic information. Likewise, we chose the R-SPIN task because it assesses not only general speech-in-noise perception abilities but also the use of high-level semantic cues when perceiving speech-in-noise. The presence of ASD symptoms have been identified within TD populations (Baron-Cohen et al., 2001) and those TD individuals who present a greater amount of ASD symptoms tend to perform similarly to individuals who are formally diagnosed with ASD on auditory (Stewart et al., 2018) and visual perception tasks (Almeida et al., 2010). Therefore, in addition to comparing ASD children with TD children, we also examined whether age-normed IQ and ASD symptoms in a larger sample of TD children predict the use of semantic information in our tasks. This offers an additional test of whether reduced semantic processing is associated with ASD characteristics.

## Method

### Participants

We recruited 29 children diagnosed with an autism spectrum disorder (ASD) (21 male; age range = 7.17 to 14.92 years, mean age = 11.18 years) from the UNLV Ackerman Center for Autism and Neurodevelopment Solutions, and 109 TD children (47 male; age range = 7.0 to 14.58 years, mean age = 9.62 years) from the general Las Vegas community. TD children had no reported personal history of neurological or developmental disorders.

All parents/caregivers reported that participants had normal hearing and provided written informed consent in accordance with the guidelines of the University’s Office for the Protection of Research Subjects for their child to participate. The children were all native English speakers and provided assent prior to testing. Diagnosis of ASD was performed using the Autism Diagnostic Observation Schedule (Second Edition, Western Psychological Services) or the Childhood Autism Rating Scale (Second Edition, Western Psychological Services), along with clinical impressions from a licensed psychologist or developmental behavioral pediatrician. UNLV Ackerman Center staff provided confirmation of diagnosis via medical records or diagnosis was performed anew by Ackerman Center staff. A total of eight children with ASD reported comorbid diagnoses. These included: language impairment and reading disability (*n* = 1), cognitive and language impairment (*n* = 1), seizures (*n* = 1), Attention deficit hyperactivity disorder (*n* = 4), auditory processing disorder (*n* = 1). Two children with ASD were excluded due to child refusal to complete some tasks. Four TD children were excluded due to not completing all tests. Final analyses thus included 27 children with ASD and 105 TD children.

A priori power analyses were performed using the program MorePower (Campbell & Thompson, 2012) to determine the sample size needed for each group for a medium effect size of n_p_^2^ = 0.13 with 80% power using a mixed-design analysis of variance (ANOVA) for the change deafness and speech-in-noise tasks. Results indicated that 27 participants per group would be needed to detect significant main effects of trial type (same, different) and change type (acoustic, semantic), and to detect significant trial type x group and change type x group interactions for the change deafness task. Additionally, 27 participants per group would be needed to detect a significant main effect of sentence type (HP, LP) and a significant sentence type x group interaction for the R-SPIN task (see below for detailed description of design). However, we recruited a large sample of TD children to ensure sufficient power for regression analyses and to precisely pair TD controls with individuals in the ASD group.

Table 1 shows key participant characteristics. To select age- and IQ-matched TD controls from the larger sample of 105 TD participants, we matched individual children in the ASD group (age range = 8.08 to 14.92 years) with 27 individual TD children who were the same age (+/- 1 year) as one of the children in the ASD group (age range = 7.58 to 14.50 years) and another group of TD children who had the same full-scale IQ (+/- 12 points) as children in the ASD group (age range = 7.25 to 14.58 years). The age-matched and IQ-matched control groups had nine participants in common. When there was more than one TD match for an ASD child, we randomly selected a TD match. Accordingly, age did not differ between the ASD (M = 134.37 months, SD = 24.20) and age-matched TD group (M = 133.22 months, SD = 23.31), *t*(52) = 0.18, *p* = .860; *d* = 0.05. Likewise, IQ did not differ between the ASD (M = 88.85, SD = 18.17) and IQ-matched TD group (M = 91.15, SD = 15.92), *t*(52) = − 0.49, *p* = .624; *d* = − 0.14. Age also did not differ between the ASD (M = 134.37 months, SD = 24.20) and IQ-matched TD group (M = 125.33 months, SD = 28.47), *t*(52) = 1.26, *p* = .214; *d* = 0.03. However, IQ did differ between the ASD (M = 88.85, SD = 18.17) and age-matched TD group (M = 104.70, SD = 14.60), *t*(52) = -3.53, *p* < .001; *d* = − 0.96. IQ Vocabulary and Matrix average sub-scores are as follows: for the ASD group (Vocabulary: M = 42.19, SD = 12.04; Matrix: M = 45.52, SD = 11.58), for the Age-matched TD group (Vocabulary: M = 53.26, SD = 11.83; Matrix: M = 52.67, SD = 9.84), and for the IQ-matched TD group (Vocabulary: M = 45.33, SD = 11.97; Matrix: M = 45.85, SD = 10.18).

**Table 1:** Participant Characteristics of ASD and Age- and IQ-matched Control Groups

Group	Sex Ratio (M/F)	Chronological Age (years)	IQ	GARS
ASD (*n* = 27)	19/8	11.19 (2.02)	88.85 (18.17)	98.44 (10.34)
TD Age-matched (*n* = 27)	19/8	11.10 (1.94)	104.70 (14.59)	55.67 (10.28)
TD IQ-matched (*n* = 27)	19/8	10.44 (2.37)	91.15 (15.92)	57.70 (15.25)

Note: Means and standard deviations (in parentheses) are presented.

### Apparatus

All participants completed the change deafness task in a quiet room using either a MacPro4.1 running Windows7 Enterprise or a HP ProBook 645 G1 computer running Windows 10, and stimuli were presented using a custom script in Presentation (Version 16.3). Sounds were presented through KidzGear headphones, Sony Professional MDR-7506 headphones, or Sennheiser HD 280 pro headphones at around 60 dB SPL. Both headphones have similar frequency responses (KidzGear, Sennheiser = 20 Hz – 20 kHz; Sony = 10 Hz – 20 kHz) and sensitivity (KidzGear = 108 dB $\pm$ 3 dB; Sony = 106 dB; Sennheiser = 117 dB). A green and red sticker was placed over the letters “S” and “D” on the keyboard and a custom Presentation script recorded participants’ keyboard presses.

Participants completed the speech-in-noise task in either a sound-attenuated booth (Industrial Acoustics Corp., Bronx, NY) using a Pentium 4 computer with a SB X-Fi sound card (Creative Technology, Ltd.), or in a quiet room using a HP ProBook 645 G1 computer running Windows 10. Stimuli was presented using a custom script in Presentation (Version 16.3). Sounds were presented through Sennheiser HD 280 pro headphones at around 60 dB SPL. The experimenter was seated in the testing room with the participant and wrote down the participants’ verbal responses for later scoring.

### Stimuli

The change deafness task was adapted from a prior paradigm (Gregg & Samuel, 2009; Vanden Bosch der Nederlanden et al., 2016). Auditory stimuli consisted of 14 unique sound types with two exemplars for each sound type (e.g., dog A and dog B, trumpet A and trumpet B, etc.) resulting in a total of 28 sounds. Male and female voices saying the syllable “ma” were included as two additional sound types to assess possible attentional biases for detecting changes that involve human voices. Five members of our lab rated 8 sounds (4 male voices and 4 female voices) based on similarity. Two male voices and two female voices with the greatest within-gender dissimilarity ratings were included in the current study.

**Fig. 1:**
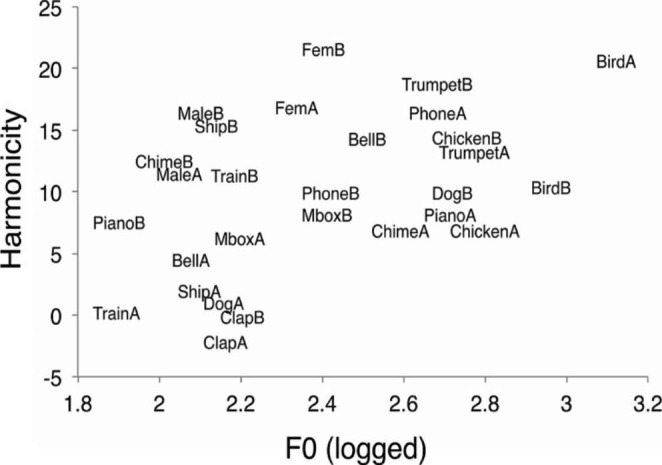
Acoustic features of sounds used in change deafness task. Harmonicity (measured in dB) and log of mean fundamental frequency (measured in Hz) for each sound stimulus included in the change deafness task. This two-dimensional space was used to calculate the Euclidian distance between sound pairs. Borrowed with permission from Vanden Bosch der Nederlanden et al. (2016), Fig. 1

To create change trials for each change-type condition (across-category, within-category, acoustically similar, and acoustically dissimilar), sound pairs were created based on Euclidean distance and superordinate category as in Gregg and Samuel (2009) (See Fig. 1). Within- and across-category sound pairs were created by pairing sounds that come from the same (within-category) or different (across-category) superordinate category. The four superordinate categories were human voice, musical instrument, animal, and environmental. To create semantic changes, we selected 14 across-category and 14 within-category sound pairs that were equated for Euclidian distance to control for acoustic similarity. For example, an across-category sound pair could include “dog A” and “phone B” with a Euclidian distance of 8.83 while its within-category counterpart could include “dog A” and “dog B” with a Euclidian distance of 8.74 (see Fig. 1). Likewise, to create acoustic changes, we selected sound pairs (all across-category), 14 of which were acoustically similar and 14 of which were dissimilar, with a Euclidian distance of 0–4 and 8–13, respectively. For example, an acoustically similar sound pair could include “Bird A” and “Female voice A” with a Euclidian distance of 2.33 and an acoustically dissimilar sound pair could include “Bird A” and “Piano B” with a Euclidian distance of 11.87. Auditory scenes were comprised of four 1s sounds with simultaneous onsets. To create the auditory scenes, three other sounds were randomly selected by a custom program in MATLAB, with the constraint that there were never two exemplars from the same basic-level sound type in any given scene.

We also used the Revised Speech Perception in Noise Task (R-SPIN) (Bilger et al., 1984) to assess comprehension of speech-in-noise. R-SPIN sentences are digital copies taken from the R-SPIN CD obtained from the University of Illinois, Department of Speech and Hearing Sciences. We selected 90 sentences (Lists 1 and 2 from the CD) that contained 45 words used once in a high-predictability (HP) sentence and once in a low-predictability (LP) sentence. For HP sentences, the target word could be predicted by the semantic cues of the sentence, whereas the target word in LP sentences could not. For example, a HP sentence would be “The dog chewed on the *bone.*” and its LP counterpart would be “Miss Black would consider the *bone.*” All sentences were presented in in multitalker babble, which remained at a constant level of 65 dB SPL while the level of the sentences varied. This gave rise to nine different signal-to-noise ratios (SNR), ranging from − 1 to 23 dB SNR in 3 dB increments, as in a previous study (Vanden Bosch der Nederlanden et al., 2020). Ten sentences were presented at each SNR.

### Procedure

IQ was measured by the Wechsler Abbreviated Scale of Intelligence – Second Edition (WASI) (Wechsler, 2011) two-subtest IQ (Vocabulary and Matrix Reasoning subtests) and raw scores were converted to age-normed full-scale IQ. Testing took place in a quiet room with the child seated across a table from the test administrator. To obtain a measurement of ASD symptom severity, all participants’ caregivers completed the Gilliam Autism Rating Scale – Third Edition (GARS-3) (Gilliam, 2014). The GARS-3 is a questionnaire that includes 58 Likert-type items about typical behaviors of the individual being rated. Items are organized into 6 sub-scales: restricted/repetitive behaviors, social interaction, social communication, emotional responses, cognitive style, and maladaptive speech. Once scored, the GARS-3 provides an autism index that ranges from 43 (unlikely probability of ASD diagnosis) to 140 (Very likely probability of ASD diagnosis).

**Fig. 2:**
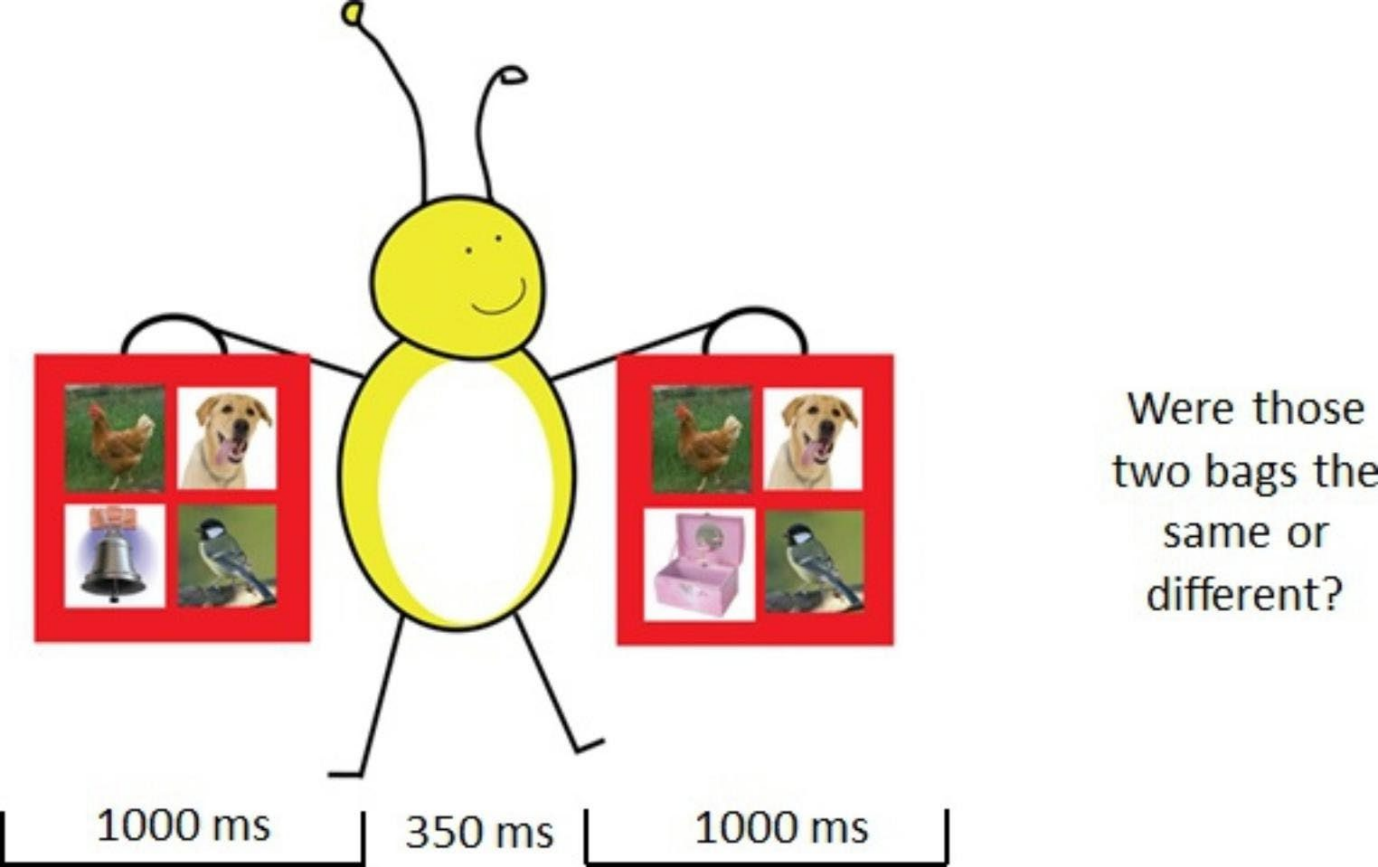
Change deafness trial example. An example of a ‘different’ change deafness trial used in the current study. Pictures of sounds were only present during the training phase, not during the test trials. Borrowed with permission from Vanden Bosch der Nederlanden et al. (2016), Fig. 1

The current study used the *one-shot paradigm* for the change deafness task, similar to our prior study (Vanden Bosch der Nederlanden et al.,^2020^). Participants were presented with two 1s auditory scenes separated by a 350 ms silent interval. After hearing the two scenes, participants indicated whether they sounded the same or different by pressing a green key for “same” and a red key for “different”. See Fig. 2 for an example of a change deafness trial. Same trials had identical sounds for both scenes, whereas on change trials one sound changed from scene 1 (S1) to scene 2 (S2) and the other 3 sounds stayed the same. There were four types of change trials (within-category, across-category, acoustically similar, and acoustically dissimilar). There were 56 change trials (14 for each change type). Across change trials, a sound could change into one of four superordinate categories: human voice, musical instrument, animal, or environmental sound. Across all change trials, the new sound in S2 was a human voice on 14 trials, a musical instrument on 16, an animal on 6, and an environmental sound on 20 trials. Grouping the change trials in this manner would reveal any automatic attentional biases for detecting changes to a particular superordinate category. We included 28 same trials to calculate false alarm rate. Altogether, participants completed a total of 84 trials across four blocks with 21 trials in each block. Participants were offered a break at the end of each block.

All participants first completed a training phase to familiarize them with the change deafness task. The training included one sample each of a same and a different trial with accompanying pictures of what sounds were in the auditory scenes. Participants then completed two easy practice trials that contained only two sounds in each auditory scene. Next, participants completed four practice trials with four sounds per scene, one from each change type: across-category, within-category, acoustically similar, acoustically dissimilar, and same. On practice trials, there were no pictures of the sounds but we did provide feedback (correct or incorrect?).

For the R-SPIN task, on each trial participants heard spoken sentences and repeated back the last word of each sentence (target word). As described above, each of the 45 words was presented once in each of two lists: once in a HP sentence and once in a LP sentence. Individual words were randomly assigned to the HP or LP context for each list, and to a given SNR level. Each word appeared at the same SNR in both lists and there were five words at each of the 9 SNR levels. Each list presented sentences in descending SNR order beginning with 23 dB SNR. All sentences were presented to the left ear only, in accordance with the typical administration of this task to only one ear (Wilson et al., 2012). Participants first completed 5 practice sentences to familiarize them with the task. The test block consisted of 5 blocks of 18 sentences. Participants were offered a break at the end of each block.

To promote sustained engagement over the course of testing, we created a visual token system where participants earned 10 stars throughout the visit to put on their star board. Once the participant earned 10 stars, they chose a prize to take home. Participants earned one star after completion of the WASI, four stars during the change deafness task, and five stars during the R-SPIN. The experimental paradigms were designed according to well-established strategies for keeping children optimally engaged in psychophysical tests (Abramov et al., 1984). For the change deafness task, participants were told a story about a yellow bug named Bugsy who was throwing a party for all his friends, in line with our previous study (Vanden Bosch der Nederlanden et al., 2020). He wanted to give all of his guests identical party bags (auditory scenes) that contained 4 sound-making toys in them. Bugsy claimed that someone had been changing the toys in the party bags so now some of them were no longer the same. The participants were asked to help figure out which party bags were the same (same trials) and which party bags were different (different trials) to solve the mystery of the toy-changing bandit.

For the R-SPIN task, participants were told to imagine they had been selected class leader during a field trip with their classmates and teacher, Mr. Scruffs. The child’s job was to listen carefully to Mr. Scruffs and repeat back the last word of each sentence to their classmates so everyone would know the field trip rules so the field trip wouldn’t get canceled. Participants were told that their classmates would be talking in the background (multitalker babble) at the same time as Mr. Scruffs (target sentence) so it might be hard to hear sometimes but to just do their best.

With consent, participants were video recorded during the assessments and experimental tasks. We later used these recordings to rate the child’s attentiveness. We counterbalanced the order of the change deafness and R-SPIN tasks.

### Data Analyses

#### Change Deafness

For all analyses described below, we conducted two separate ANOVAs for each control group, one comparing the ASD group to the age-matched TD control group, and the other comparing the ASD group to the IQ-matched control group. To evaluate the presence or absence of change deafness in ASD and TD groups, we first submitted error rates to a mixed model analyses of variance (ANOVA) with trial type (same, different) as the within-participants factor and group (ASD, TD) as the between-participants factor.

For the remaining analyses we calculated d’ scores to account for response bias. To examine change the influence of semantic information on detection ability, we entered d’ scores for semantic change trials into mixed model ANOVAs, with semantic change type (across, within) as the within-participants factor and group (ASD, TD) as the between-participants factor. To examine the influence of acoustic differences on change detection, we entered d’ scores for acoustic-change trials into mixed model ANOVAs, with acoustic change type (dissimilar, similar) as the within-participants factor and group (ASD, TD) as the between-participants factor. We also wanted to examine the extent to which listeners use acoustic or semantic information when detecting changes. If listeners rely more on semantic than acoustic information, we should expect higher error rates for the within category semantic change trials than for the acoustically similar change trials, even though the average Euclidian distance would predict the opposite (see Stimuli). Thus, we submitted d’ scores on these trials to a mixed model ANOVA with change type (within, similar) as the within-participants factor and group (ASD, TD) as the between-participants factor. Lastly, to investigate whether TD and ASD children display an attentional bias to detect changes from socially relevant semantic categories, d’ was calculated for each superordinate category change type (human voices, environmental sounds, musical instruments, animal sounds). These values were entered into a mixed model ANOVA with group (ASD, TD) as the between-participants factor and category change type (human voices, environmental sounds, musical instruments, animal sounds) as the within-participants factor.

#### RSPIN

For the speech-in-noise task (RSPIN), percent correct for each speech-to-noise ratio (SNR) and for each sentence type was calculated. To test for possible differences in the use of semantic information across the 9 SNRs between the groups, these values were entered into a 2 × 9 × 2 mixed model ANOVA, with group (ASD, TD) as the between-participants factor and SNR level (-1, 2, 5, 8, 11, 14, 17, 20, 23) and sentence type (high predictability, low predictability) as within-participants factors.

#### Relationships Among IQ, ASD Symptoms, and the Use of Acoustic and Semantic Information

To understand whether overall IQ and total GARS scores could predict the use of acoustic and semantic information in TD children, four regressions were performed. All regressions included TD children’s IQ and GARS scores as the predictors. The dependent variables for the regressions were as follows: (1) difference in performance between within- and across-category changes from the change deafness task (2) difference in performance between the dissimilar and similar acoustic changes from the change deafness task (3) difference in performance between the high- and low-predictability sentences for the lowest SNR’s (5, 2, -1) from the speech-in-noise task (4) difference in performance between the high- and low-predictability sentences of all SNR’s from the speech-in-noise task. A total of one-hundred and five TD children were included in these analyses (45 male; age range = 7 years 0 months to 14 years 7 months, mean age = 9 years 6 months, Mean IQ = 105, Mean GARS = 57).

#### Attentiveness Ratings

Recorded videos of the participants completing the WASI, change deafness, and RSPIN sessions were coded by seven raters for attentiveness. To evaluate inter-rater reliability, twenty videos were randomly chosen for all seven trained raters to code. The remaining videos were randomly assigned such that each rater got an equal number of videos and each video was coded twice by two separate raters. Raters assigned an attentiveness rating at one-minute intervals using a 0–4 scale (Koegel & Egel, 1979), with larger scores meaning greater attentiveness.

To evaluate inter-rater reliability an intraclass correlation coefficient (ICC) was computed using the average attentiveness scores from the twenty videos that were coded by all raters. ICC estimates and their 95% confidence intervals were calculated using SPSS statistical package version 27 based on a mean rating (*k* = 7), absolute-agreement, two-way mixed model effects. Results revealed an intraclass correlation coefficient of 0.88 with a 95% confidence interval of 0.73 − 0.95. To test for possible differences in attentiveness between groups, the averaged attentiveness score across all tasks was computed for each participant, resulting in one attentiveness score per participant. These scores were entered into an independent sample t-test separately for each data set (ASD vs. age-matched and ASD vs. IQ-matched). Due to not all participants being video recorded, the randomly assigned age- and IQ-matched control groups resulted in a total of 14 and 18 participants being included for these analyses, respectively. A total of 21 participants from the ASD group were included.

## Results

### Change Deafness

**Fig. 3:**
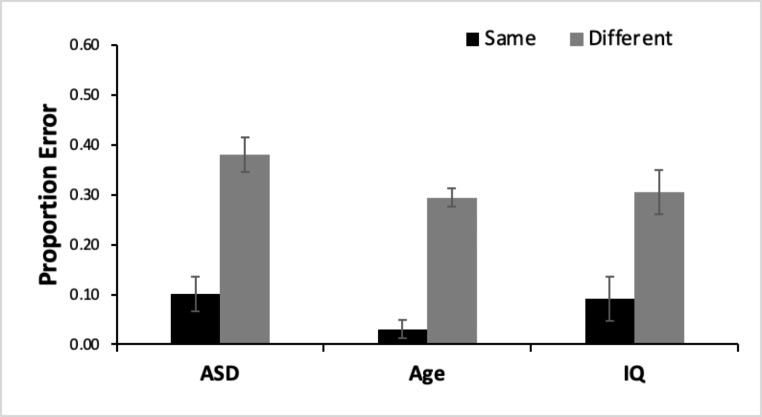
Presence of Change Deafness. Percent error for same and different trials for children with ASD, age-matched controls (age), and IQ-matched controls (IQ). Error bars represent within-participant 95% confidence intervals (Cousineau, 2005)

As seen in Fig. 3, all groups exhibited change deafness with higher error rates on different trials than same trials for both the ANOVA using age-matched controls, *F*(1, 52) = 178.89, *p* < .001, n_p_^2^ = 0.77, as well as the ANOVA using IQ-matched controls, *F*(1, 52) = 72.27, *p* < .001, n_p_^2^ = 0.58. There was a significant main effect of group in the analysis with age-matched controls, *F*(1, 52) = 10.47, *p* = .002, n_p_^2^ = 0.17, but not in the analysis with IQ-matched controls, *F*(1, 52) = 2.15, *p* = .148, n_p_^2^ = 0.04, indicating that age-matched TD controls but not IQ-matched controls had lower error rates (i.e. less change deafness) than children with ASD. There was no interaction between trial type and group (age-matched: *F*(1, 52) = 1.58, *p* = .692, n_p_^2^ = 0.003, IQ-matched: *F*(1, 52) = 1.31, *p* = .257, n_p_^2^ = 0.03). Effect sizes suggest that all ASD and TD control groups exhibited change deafness similarly.

**Fig. 4:**
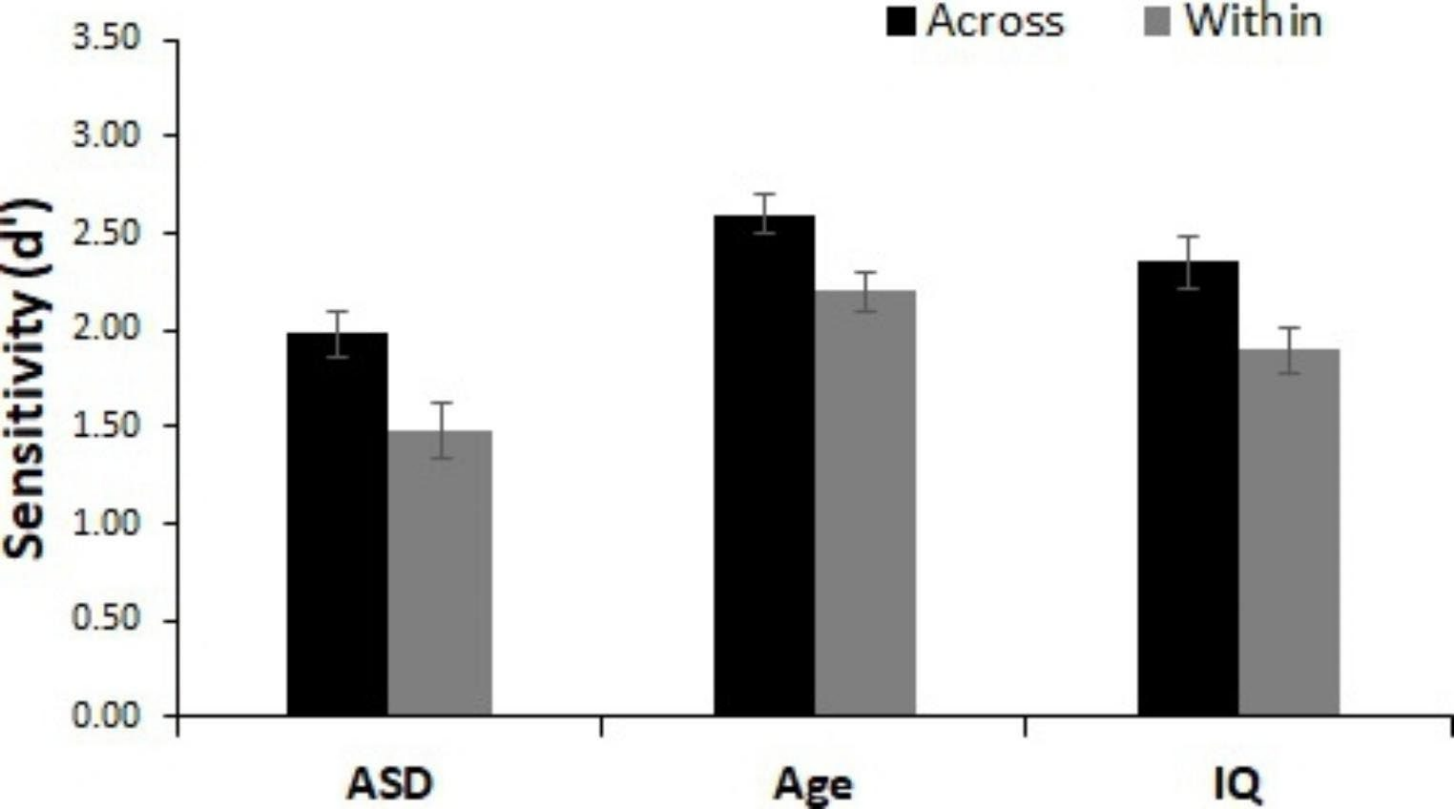
Performance for Semantic Change Trials. d’ scores for across- and within-category changes for all groups. All groups utilize semantic information such that within-category changes were more difficult to detect than across-category changes. Error bars represent within-participant 95% confidence intervals (Cousineau, 2005)

As depicted in Fig. 4, there was a significant main effect of semantic change type, indicating worse change detection for within-category changes than for across-category changes (age-matched: *F*(1, 52) = 41.57, *p* < .001, n_p_^2^ = 0.44, IQ-matched: *F*(1, 52) = 38.41, *p* < .001, n_p_^2^ = 0.43). We observed a significant main effect of group for the analysis with age-matched controls, *F*(1, 52) = 12.73, *p* < .001, n_p_^2^ = 0.20, but not for the analysis with IQ-matched controls, *F*(1, 52) = 3.02, *p* = .088, n_p_^2^ = 0.06, suggesting the ASD group performed significantly worse overall than the age-matched TD control group but not the IQ-matched TD control group. There was no interaction between semantic change type and group (age-matched: *F*(1, 52) = 0.621, *p* = .434, n_p_^2^ = 0.01, IQ-matched: *F*(1, 52) = 0.11, *p* = .745, n_p_^2^ = 0.002).

**Fig. 5:**
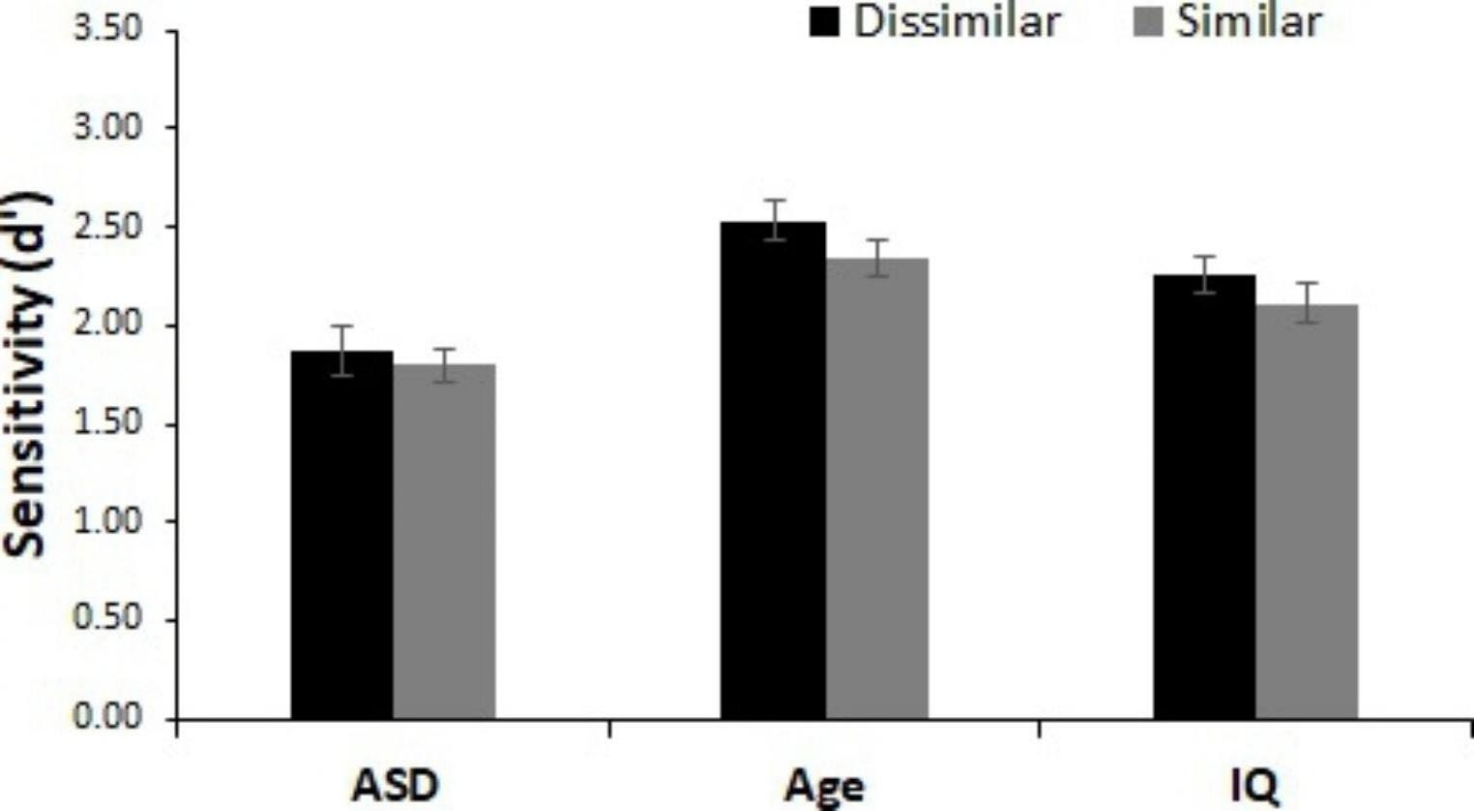
Performance for acoustic change trials. d’ scores for acoustically similar and dissimilar changes for all groups. All groups utilize acoustic information such that acoustically similar changes were more difficult to detect than acoustically dissimilar changes. Error bars represent within-participant 95% confidence intervals (Cousineau, 2005)

As seen in Fig. 5, there was a significant main effect of acoustic change type, indicating poorer detection of similar acoustic changes than dissimilar acoustic changes (age-matched: *F*(1, 52) = 5.68, *p* = .021, n_p_^2^ = 0.10, IQ-matched: *F*(1, 52) = 4.21, *p* = .045, n_p_^2^ = 0.08). there was again a significant main effect of group in the analysis with age-matched controls, *F*(1, 52) = 9.22, *p* = .004, n_p_^2^ = 0.15, but not with IQ-matched controls, *F*(1, 52) = 2.25, *p* = .140, n_p_^2^ = 0.04. There was no interaction between acoustic change type and group (age-matched: *F*(1, 52) = 1.24, *p* = .270, n_p_^2^ = 0.02, IQ-matched: *F*(1, 52) = 0.48, *p* = .490, n_p_^2^ = 0.01).

**Fig. 6:**
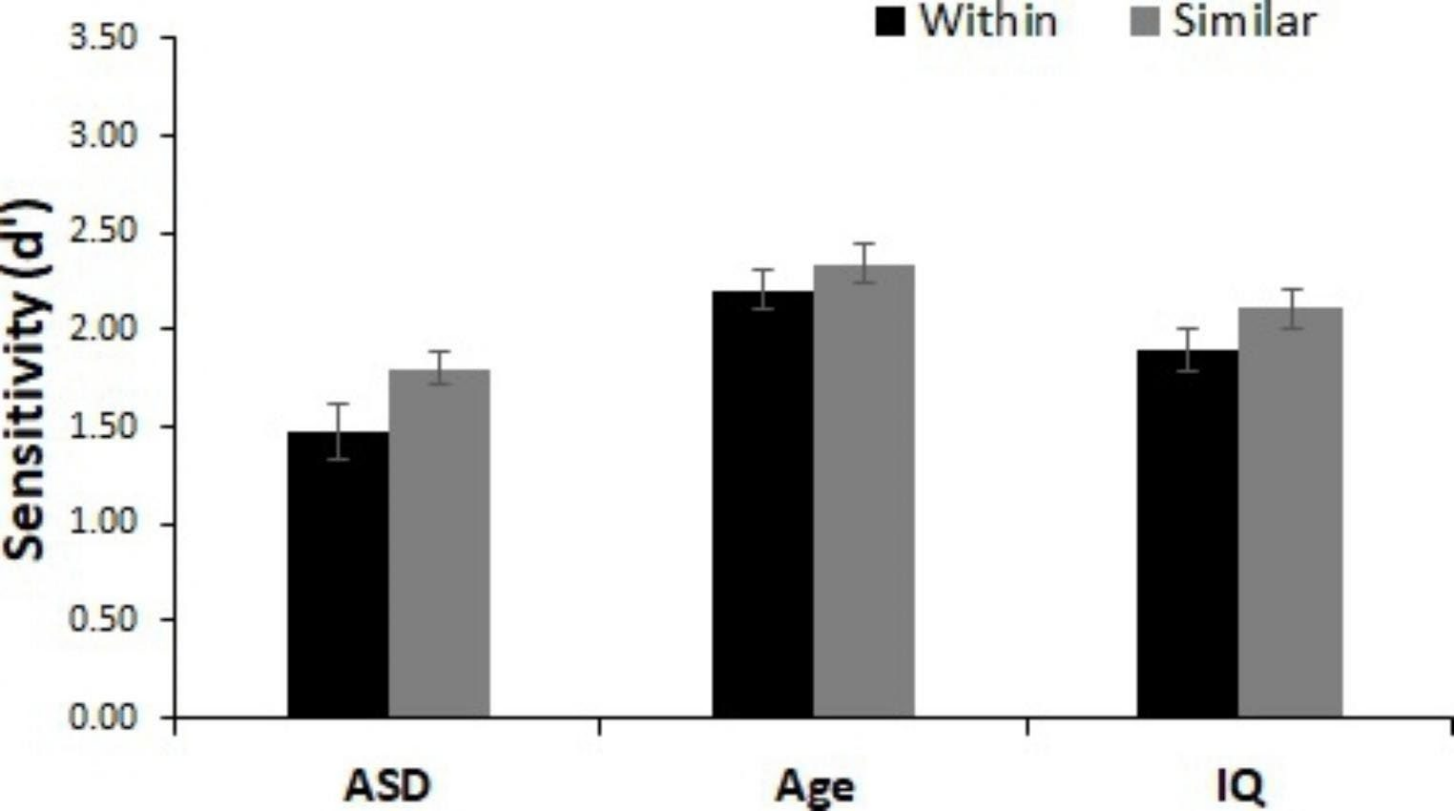
Magnitude of the use of semantic vs. acoustic information. d’ scores for within-category and acoustically similar changes for all groups. All groups utilize semantic information to a greater degree than acoustic information such that within-category changes were more difficult to detect than acoustically similar changes. Error bars represent within-participant 95% confidence intervals (Cousineau, 2005)

As seen in Fig. 6, all children were better at detecting within category semantic changes than similar acoustic changes (age-matched: *F*(1, 52) = 13.85, *p* < .001, n_p_^2^ = 0.21, IQ-matched: *F*(1, 52) = 16.62, *p* < .001, n_p_^2^ = 0.24. There was again a significant main effect of group in the analysis with age-matched controls, *F*(1, 52) = 14.82, *p* < .001, n_p_^2^ = 0.22, but not in the analysis with IQ-matched controls, *F*(1, 52) = 3.45, *p* = .069, n_p_^2^ = 0.06. There was no interaction between change type and group (age-matched: *F*(1, 52) = 2.23, *p* = .141, n_p_^2^ = 0.04, IQ-matched: *F*(1, 52) = 0.66, *p* = .421, n_p_^2^ = 0.01).

**Fig. 7:**
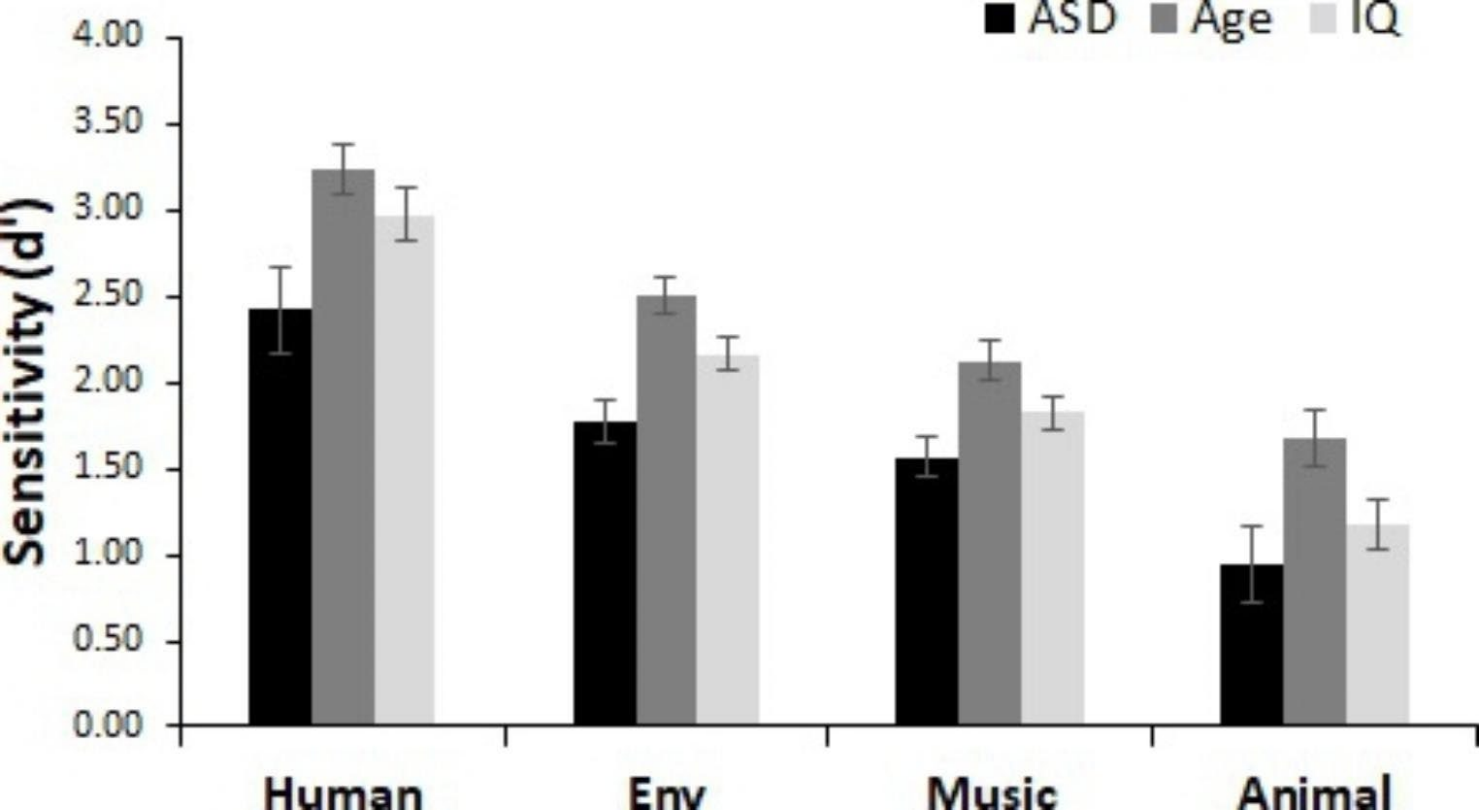
Performance for semantic categories. Sensitivity (d’) for each semantic category change type for all groups. All groups displayed the greatest sensitivity to detect changes that involve the human voice, followed by environmental sounds, then musical instruments, then animal sounds. Error bars represent within-participant 95% confidence intervals (Cousineau, 2005)

As shown in Fig. 7, detection performance did vary by change type (i.e., voice, environment sounds, musical instruments, animal sounds). We observed a significant main effect of category change type (age-matched: *F*(3, 156) = 85.00, *p* < .001, n_p_^2^ = .62, IQ-matched: *F*(3, 156) = 101.69, *p* < .001, n_p_^2^ = .66). Planned comparisons revealed that all categories were significantly different from one another (*p*’s < .001) such that all groups were best at detecting changes that involved the human voice (d’ scores: age-matched = 2.82, IQ-matched = 2.69) followed by environmental sounds (age-matched = 2.13, IQ-matched = 1.96), musical instruments (age-matched = 1.84, IQ-matched = 2.69), and animal sounds (age-matched = 1.31, IQ-matched = 1.06). There was a main effect of group for the analysis of age-matched controls, *F*(1, 52) = 15.76, *p* < .001, n_p_^2^ = 0.23, but not IQ-matched controls, *F*(1, 52) = 2.30, *p* = .135, n_p_^2^ = 0.04, suggesting that age-matched but not IQ-matched controls were better overall at change detection than children with ASD. There was no significant interaction between group and category change type (age-matched: *F*(1, 52) = 0.64, *p* = .593, n_p_^2^ = 0.01, IQ-matched: *F*(3, 156) = 1.19, *p* = .315, n_p_^2^ = 0.02).

#### RSPIN

**Fig. 8:**
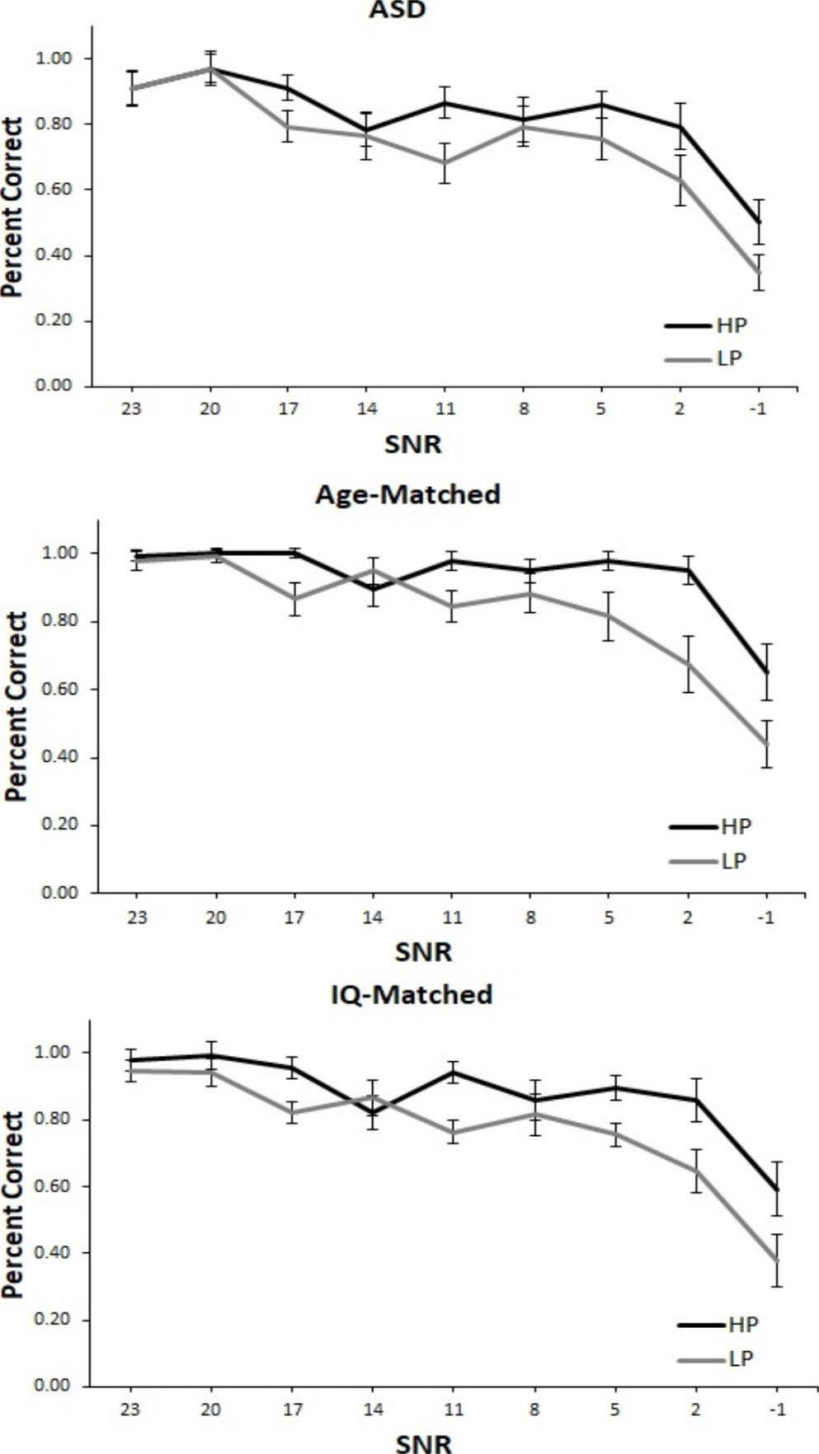
Performance for RSPIN task. Percent correct for high-predictability (HP) and low-predictability (LP) sentences for each signal-to-noise ratio (SNR) for the (A) ASD, (B) age-matched, and (C) IQ-matched groups. The ASD group performed worse overall relative to age- and IQ-matched controls. All groups utilized semantic context such that performance was higher for HP relative to LP sentences

Figure 8 depicts the results for the RSPIN task. All participants performed better at higher SNR levels, as shown by a significant main effect of SNR level (age-matched: *F*(8, 416) = 108.06, *p* < .001, n_p_^2^ = 0.68, IQ-matched: *F*(8, 416) = 98.65, *p* < .001, n_p_^2^ = 0.65). There was a main effect of group in the analysis with age-matched controls, *F*(1, 52) = 3527.50, *p* < .001, n_p_^2^ = 0.98, but not in the analysis with IQ-matched controls, *F*(1, 52) = 1.54, *p* = .220, n_p_^2^ = 0.03, suggesting that overall, age-matched but not IQ-matched TD children outperformed children with ASD. SNR level did not interact with group (age-matched: *F*(8, 416) = 1.74, *p* = .087, n_p_^2^ = 0.03, IQ-matched: *F*(8, 416) = 0.73, *p* = .666, n_p_^2^ = 0.01). All participants were better at the task when sentences were predictable, as shown by a significant main effect of sentence type (age-matched: *F*(1, 52) = 88.60, *p* < .001, n_p_^2^ = 0.63, IQ-matched: *F*(1, 52) = 77.15, *p* < .001, n_p_^2^ = 0.60). However, sentence type did not interact with group (age-matched: *F*(1, 52) = 1.02, *p* = .317, n_p_^2^ = 0.02, IQ-matched: *F*(1, 52) = 0.95, *p* = .334, n_p_^2^ = 0.02). Participants appeared to rely more on semantic content at lower than at higher SNR levels, as shown by the interaction between sentence type and SNR level (age-matched: *F*(8, 416) = 9.94, *p* < .001, n_p_^2^ = 0.16, IQ-matched: *F*(8, 416) = 8.12, *p* < .001, n_p_^2^ = 0.14). The 3-way interaction between SNR level, sentence type, and group was not significant (age-matched: *F*(8, 416) = 1.07, *p* = .380, n_p_^2^ = 0.02, IQ-matched, *F*(8, 416) = 0.48, *p* = .872, n_p_^2^ = 0.01.

#### Predicting Use of Semantic and Acoustic Information from IQ and ASD Symptoms

**Fig. 9:**
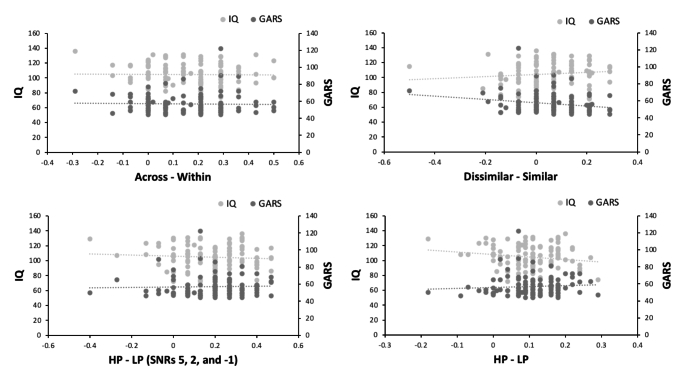
GARS, IQ, and perceptual performance correlations in TD Children. Scatterplots that show the relationship between IQ, GARS and across category – within category changes for the change deafness task (across – within), high-predictability – low predictability sentences for all SNR’s for the speech-in-noise task (HP – LP), high-predictability – low-predictability sentences for SNR’s 5, 2, and − 1 for the speech-in-noise task (HP – LP (SNRs 5, 2, -1)), and acoustically dissimilar – acoustically similar changes for the change deafness task (dissimilar – similar)

Figure 9 presents the results of all four regressions using the four dependent measures described above (Data Analysis section). In the change deafness task, IQ and GARS scores did not predict the use of acoustic information, R^2^ = − 0.033, F(2, 104) = 2.80, *p* = .065, nor semantic information, R^2^ = − 0.019, F(2, 104) = 0.04, *p* = .959. They also did not predict the use of semantic information during speech-in-noise perception (All SNR’s: R^2^ = 0.009, F(2,104) = 1.47, *p* = .236; Low SNR’s: R^2^ = − 0.014, F(2, 104) = 0.294, *p* = .746).

#### Attentiveness Ratings

Attentiveness scores were comparable for the ASD (M = 3.18, SD = 0.52) and age-matched control group (M = 3.36, SD = 0.49), *t*(33) = -1.05, p = .302; d = 0.5, as well as for the IQ-matched control group (M = 3.42, SD = 0.39), *t*(37) = -1.56, p = .127; d = 0.5.

## Discussion

Overall, our findings suggest that compared with their age-matched peers, children with ASD showed poorer performance on the change deafness task and poorer speech-in-noise perception, although they performed similarly to IQ-matched peers. Nevertheless, children with ASD were no less likely to rely on semantic and acoustic information in the change deafness task, nor were they less likely to use semantic and acoustic information in the speech-in-noise task. Finally, children with ASD and TD children showed similar attentional biases towards human voices.

In the change deafness task, children showed better performance for changes that were incongruent with a semantic category, and they were best at detecting changes that involved the human voice. These results replicate previous findings within typically developing adults and children (Gregg & Samuel, 2009; Vanden Bosch der Nederlanden et al., 2016) and extend these findings to children with ASD. Similarly, during the speech-in-noise task, both TD and ASD children were better able repeat the last word in a sentence when the SNR level was higher and when the sentence was predictable, suggesting both groups used acoustic and semantic information in this task. This is consistent with prior behavioral research showing unimpaired processing of semantic information in individuals with ASD during matching (McCleery et al., 2010) and priming tasks (Kamio & Toichi, 2007) that involve individually presented pictures, spoken words, and semantically meaningful non-speech sounds. This suggests that both TD and ASD children can encode sounds based on semantic knowledge and use this information to detect auditory changes and to understand speech in noisy situations. It also suggests that children with ASD have the capacity to form meaningful taxonomies of everyday sounds and organize them based on this knowledge.

In contrast to prior work, we found no evidence that children with ASD have enhanced processing of acoustic features, such as pitch and harmonicity in the change deafness task given that group did not interact with the amount of acoustic change. Similarly, there was no interaction between group and signal-to-noise ratio in the speech-in-noise task, suggesting similar ability to use acoustic information to segregate the target sentence from the background noise. Although some studies suggest that children with ASD have enhanced processing of pitch for individually presented pure tones (Bonnel et al., 2003; Heaton, 2005; Heaton et al., 1999; Mayer et al., 2016; O’Riordan & Passetti, 2006) and speech stimuli (Heaton et al., 2008; Jarvinen-Pasley & Heaton, 2007; Jarvinen-Pasley, Pasley et al., 2008; Jarvinen-Pasley, Peppe, et al., 2008), children with ASD in our study did not place more weight on acoustic than semantic information. Given that the current study is the first to present complex auditory scenes with multiple simultaneous sound sources to children with ASD, it is possible that enhanced acoustic processing does not benefit listeners with more complex, multi-source stimuli in tasks such as these.

Another interesting finding was that children with ASD, like TD children, displayed an attentional bias to detect changes that involve the human voice. Compared to TD children, children with ASD have been shown to orient less to social stimuli, such as hands clapping or their name being called, (Dawson et al., 1998) and they prefer orienting to speech-derived noise than to maternal or child-directed speech (Klin, 1991; Kuhl et al., 2005). These studies typically use a head-turn/orienting procedure and semantically meaningful speech stimuli. Here, rather than orienting or approaching stimuli, children were simply asked to detect changes. Likewise, the human voice stimuli did not include semantically meaningful speech. Instead, we used male and female voices with speech-like phrasing but using the syllable “ma” instead of individual words to avoid accessing verbal memory or semantic representations. The ability to detect changes to social stimuli during visual change blindness tasks have been investigated in individuals with ASD. Smith & Milne (2009) found that children with ASD detected social changes that occurred to people just as well as changes involving inanimate objects, similarly to TD children. Kikuchi et al. (2009) found that TD children were faster at detecting changes to human faces relative to non-social changes while children with ASD detected the social and non-social changes equally fast. The current study did not measure reaction time but this method may reveal more subtle differences between TD children and those with ASD.

To our knowledge, this study is the first to investigate the use of semantic information during speech-in-noise perception in TD and ASD children. Children with ASD performed worse than age-matched but not IQ-matched controls; however, all groups similarly utilized semantic context. Previous research has shown impaired speech-in-noise perception in individuals with ASD (Alcantara et al., 2004, 2012; Groen et al., 2009). Here, we find that children with ASD only perform worse than age-matched controls but similarly to IQ-matched controls.

Across both tasks, children with ASD performed more poorly than age-matched but similarly to IQ-matched TD children. This suggests that lower IQ may drive poorer performance on the change deafness task, regardless of ASD diagnosis. Other factors could also influence performance in the change deafness task, such as the capacity to process multiple objects (Gregg et al., 2017), attention (Irsik et al., 2016), or auditory short-term memory (Vanden Bosch der Nederlanden et al., 2020). In the speech-in-noise task, poorer performance might also be related to working memory or deficits in temporal processing. Indeed, temporal processing is correlated with speech-in-noise perception (Bhatara et al., 2013), and temporal processing deficits have been reported in individuals with ASD (Bhatara et al., 2013; Boets et al., 2015; Foss-Feig et al., 2017). Speech-in-noise perception has also been related to working memory capabilities for phonological sounds (Akeroyd, 2008) and frequency information (Lad et al., 2020). Future research should include assessments of acoustic processing and cognitive abilities to investigate which factors are related to change deafness and speech-in-noise perception in TD and ASD children.

Lastly, IQ and ASD symptoms did not predict how likely TD children were to use acoustic or semantic information during either task. Eberhardt and Nadig (2018) found that structural language ability, not nonverbal IQ or ASD diagnosis, was a significant predictor of the use of semantic context during tasks requiring the identification of homonyms and the completion of ambiguous sentences. Here, overall IQ and overall GARS scores were used as predictors. It is possible that separating IQ into verbal and nonverbal abilities or using a more sensitive measure of specific language skills could predict the use of acoustic or semantic information during the change deafness task or during speech-in-noise perception.

As described above, prior research provides conflicting evidence regarding the question of whether children with ASD exhibit impairments in using semantic context. Compared to TD children, those with ASD have difficulty performing visual language tasks that require them to read homograph-containing sentences aloud (Frith & Snowling, 1983; Happé, 1997; Jolliffe & Baron-Cohen, 1999; Lopez & Leekam, 2003), and they have difficulty remembering lists of words that are semantically related or unrelated (Tager-Flusberg & Anderson, 1991), or making contextual inferences during reading comprehension (Norbury & Bishop, 2002). However, other studies report that when ASD children have typical receptive and expressive language abilities, they perform similarly to TD children at identifying homonyms (Melanie Eberhardt & Aparna Nadig, 2018). Although we did not specifically assess language abilities, our findings are in line with the notion that impaired use of semantic information is not universal in individuals with ASD.

### Limitations

One limitation of the current study is the somewhat modest sample size of 27 children with ASD, and the same sample sizes of age- and IQ-matched TD children. Larger sample sizes would enable more power to detect smaller effects that may be present, such as possible subtle differences in how much the three groups use semantic information. A larger sample size of ASD children would also enable us to correlate GARS scores with semantic processing within that group; we did not observe such correlations within the TD group perhaps due to range restriction of GARS scores in TD children. Likewise, testing much more than 100 TD children would enable detection of smaller correlations of semantic processing with GARS and IQ scores, more complex regression models that break down IQ scores into verbal and non-verbal components. Such a sample would also make it possible to select totally separate groups of age-matched and IQ-matched controls—including matching separately for verbal and non-verbal IQ—which would allow a more powerful omnibus ANOVA model that includes all groups of participants.

A second limitation is that we tested relatively high-functioning children with ASD, due to the requirements to perform difficult perceptual tests. Future studies that test children with a wider range of intellectual ability might reveal more robust differences in the use of semantic information during auditory segregation tasks, such as those used in the current study. A third limitation is the uneven number of stimulus types (human, animal, music, environmental) used in the change deafness task. The present study used only one type of vocal stimulus and did not equate the number of stimuli across categories, but recent work with typically developing listeners produced the advantage for human voice stimuli using equal stimulus numbers for each category, suggesting this effect is robust (Vanden Bosch der Nederlanden, Rubio-Garcia, Clarkson, & Snyder, 2020). A final limitation of the current study is that prevailing theories of perceptual processing in ASD such as WCC and EPF were not adequate for making fine-grained predictions about performance on the naturalistic tasks we used. Future studies might benefit from more precise theoretical approaches to make detailed predictions based on quantitative models of semantic processing in ASD that can then be tested in measures of human perception and neurophysiology (Robertson & Baron-Cohen, 2017).

### Summary and Conclusions

Our study found that children with and without ASD use acoustic and semantic information when attempting to detect changes between two complex auditory scenes, and they also use semantic context when perceiving speech in the presence of background noise. Unlike prior studies and in contrast to current theories of ASD, we found no evidence for enhanced acoustic processing in children with ASD, and unimpaired use of semantic context across two separate tasks using speech and non-speech sounds. Current theories of sensory and cognitive processing in ASD can be strengthened by further investigating the influence of different phenotypes on the use of acoustic and semantic information across a variety of tasks that range in task demands and stimulus complexity.
